# Women and Sanitation*Read before the Wharton-Jackson County Medical Society, June, 1908, at a special Sanitary meetihg.

**Published:** 1909-03

**Authors:** J. M. Andrews


For Texas Medical Journal.

### Women and Sanitation.*

BY MRS. J. M. ANDREWS.

We have from history, divine, the account of the creation of the earth, and through successive steps the passing from mineral to vegetable and to the animal kingdoms; from the crystal to the cell; from matter to mind, and from the material to the immaterial.

*Read before the Wharton-Jackson County Medical Society, June, 1908, at a special Sanitary meetihg.

Then, when from chaos to complete creation, how in the light of the newborn sun the multiplied forms of earth sang songs triumphant. For on the sixth day had God created man to rule over all things hitherto created. His dominion extended over the fish of the sea, and over the fowls of the air, and over the cattle, and over all the earth, and over every creeping thing.

His rule was worldwide; his power, if trained, unlimited.

Alone, could he accomplish this stupendous task? Was this stupendous responsibility too great? "For Adam there was not yet found a helpmeet," and the Lord administered unto Adam an anesthetic which caused him to fall into a deep sleep, and as he slept a rib was taken from his side from which woman was created.

It is significant that she was taken from man's side to indicate she is his equal, and also that he should protect her, and from a sentimental point of view from his left side nearest his heart that he should love her. Then God said unto them, "Replenish the earth and subdue it." Thus, by necessity and command divine, woman is han's co-ordinate, his companion, his helpmeet, his equal.

Some logicians and philosophers have spun subtle webs of thought apropos the superiority of man's intellect to that of woman's. Yet the pages of history are replete with evidence of her ability to match intellectual and moral swords with him in his every undertaking.

Notwithstanding the ages have suppressed her individualism, and ironclad customs tended to reduce her abilities and to suppress her intellectual activities, nevertheless, whenever chance has made opportunity possible, woman has proven his match in prowess and ability in initiative and executive domain.

Since the time when Adam's rib was removed and so mysteriously fashioned into a subtle human being called woman she has been busy with her many duties. Even the hush of darkness which enthralled the earth for a thousand years came and went with woman living, acting, still doing her part of the world's work in her own patient way.

Let us for a moment look retrospectively on some things she has undertaken; things that woman, individually, has done.

When the pitch of battle is on and men fall wounded and bleeding we find woman, brave and true, holding aloft the battered banner and inspiring the depleted ranks of her country's deputies to splendid victory. After the smoke has cleared away, we find her with a "heart courageous" and a patient hand going about the battlefield tenderly administering to the helpless forms of the fallen heroes. What instance of noble daring outdistances that of the brave Carthaginian women who, rather than surrender their city to the Roman foe, tore out their hair and braided it for bow strings that Carthage might be saved; or that of the French peasant girl who, when English victory followed victory, thrust herself to the front of battle and saved the French nation from permanent defeat? Who but does not know that the victories of Napoleon were the victories of the gentle Josephine or that of Marie Louise, both of whom were fountains of inspiration to the military genius and great commander.

Coming down to late history, we find woman's deeds of valor and her splendid achievements multiplied in a thousand fields of endeavor; her talents have been given opportunity to broaden and her activities permitted to expand.

Along the lines of needed reform she has ever led the vanguard of intellectual and social forces and has made good every responsibility assumed.

Notwithstanding this, our age is one of inventions and discoveries, evidences can plainly be detected of a new era. A new movement of human society has begun.

In the future the development and conservation of the social forces and the social sense will form a more and more important function of civilization's trend.

Real progress will come through the organization and co-operation of the individual unit of which society is composed. This is proving itself in a number of ways. The organization of the physicians, the teachers, the lawyers, the ministry, the merchants, the tradesmen and the workmen. The organization of the women's clubs, such, for instance, as the literary, the civics and the mothers' clubs, all bespeak the awakening of the social consciousness. The complete creation and co-operation of all these forces is the final goal.

Since by organization and co-operation most good may be accomplished, women banded together in clubs and federations of clubs become a potent factor in securing needed reforms, such as educational, political, social and sanitary.

Matters of public health and sanitation rest more with women than with men. Surely, then, there is need for women's clubs to come to the rescue of the health conditions of our towns and cities. It is admitted that the nature of woman is powerful in its influence. Then, since "in unity there is strength," the influence of many women, in form of women's clubs, is infinitely greater and broader in its scope than that wielded individually.

The clubs through committees enter the convention halls from the village schoolhouse to the nation's capital, wielding such an influence that many needed reforms have come about, yet there is a crying need for other reforms, and better laws. Statistics show that thousands of dollars have been spent to stamp out cholera among swine, but not one dollar was ever voted for eradicating tuberculosis among human beings. Vast sums of money are spent in saving the lives of trees from the attacks of beetles and in warning farmers against blights afflicting potato plants and in exterminating parasitic growths that prey on fruit trees. In fact, the Department of Agriculture has expended during the last ten years over forty-six millions of dollars. But not a wheel of official machinery was ever set in motion for the alleviation or cure of the disease of the lungs, heart or kidneys, which carry off over six million of our population in every ten years, and the event is accepted by the American people with a resignation equal to that of a Hindoo who in the midst of indescribable filth calmly awaits the day of cholera.

The logic that justifies an annual appropriation of $2,000,000 for a life-saving service against the accidents of sea should justify protection against accidents of disease and death. These are facts both startling and true and must be considered as seriously by the club woman as by the politician.

It is important to note that in securing reforms and appropriations, women's clubs have, for some time past, played an important part and in the future greater accomplishments may be expected.

Woman, having wisdom born of the homely affairs of life and a sympathy nurtured in the environments of her children, will some day, through the influence of her club, bring about such sweeping sanitary changes that the world will "sit up and take notice" of her wonderful achievements.

Let us as club women keep ever in mind that sanitation as well as "charity" begins at home, and that the community can be no better than the individual units which compose it as public health and sanitation can be no better than the average private health and sanitation, and as the mistress of the house is the mistress of all the arrangements and the cleanliness of the home, the cooking, the laundry and the home environments, so the test of the average health of the community will depend largely upon the average test of the home.

In woman's clubs definite plans are mapped out, thereby imparting strength of purpose and a feeling that we are really doing something which is the warrant of content and cheerfulness. The club makes better mothers, better housekeepers, better companions, better politicians and better sanitarians. Hence the club woman, as the counselor and sympathizer is man's natural, his logical ally in matters of public health.

Any policy of public health and sanitation which seeks to eliminate her in its promotion is doomed to defeat. On the other hand, any policy of public health and sanitation which enlists her sympathy and co-operation is most certain, most sure of success.

In the beginning of the campaign for a more sanitary Texas, the State Health Officer recognized the importance of the club's influence, and issued an appeal through the press to the women's clubs of the State, caling upon them to enlist in the great battle for health.

All over the State they answered the call, and in many different ways took up the work, each club undertaking that which seemed most needed. Some observed a "clean-up" day while others formed committees who went to the city council and demanded the passage of certain ordinances pertaining to public health and in every instance obtaining a certain measure of success. Verily, the influence of the woman's club has but just begun. We are yet in the morning of evolution, the morning of more glorious years, Well, indeed, may we anticipate a glorious future for posterity if we, the living men and women will do our duty. Science assures that the world is yet young. It assures that a hundred thousand generations of men and women may yet come and go ere the earth will cease to be habitable to members of our species; ere the light of the sun will grow pale, or its glory fade.

Then who can say but that the accumulated wisdom of all the ages that will then be possessed by the men and women of that far remote future will enable them to hold their own amid the wreck of planets or the decay of suns.

Who can place a limit of development to intelligence or define the bounds of nature's hidden powers? But it is not for us to speculate upon the problems that will confront those distant voyagers who will have arrived at the mouth of the "river of time." Who live in the morning of social evolution have not to make preparations for the night; we are yet children of the morning and have the morning's work to do.

The voices of the suffering multitudes living, the voices of the untold myriads yet to be, call us to our duty.

Let us, in the vigor of our youth, in the consciousness of our responsibility, emboldened by the achievements of humanity's past, encouraged by the prospects of the future, answer to the call.

In behalf of the New Century Club of Wharton, I assure the physicians of the county and district, and of the State, that in matters pertaining to public health and sanitation, they have the Club's hearty co-operation and support.

In this pledge I feel confident that I voice not only the New Century, but the Mutual Improvement and the Mothers' Club of Wharton. Yea, I may as well add all women's clubs of the Lone Star State join in one great federated band, standing ever ready and willing to extend to the health officers of Texas in their fight for sanitary improvements the glad, the helping hand.

